# Oxidative damage and DNA repair in desiccated recalcitrant embryonic axes of *Acer pseudoplatanus* L.

**DOI:** 10.1186/s12870-021-03419-2

**Published:** 2022-01-19

**Authors:** Beata P. Plitta-Michalak, Alice A. Ramos, Piotr Pupel, Marcin Michalak

**Affiliations:** 1 Department of Plant Physiology, Genetics and Biotechnology, University of Warmia and Mazury in Olsztyn, Oczapowskiego 1A/103, 10-719 Olsztyn, Poland; 2 Institute of Biomedical Sciences Abel Salazar (ICBAS), University of Porto (U. Porto), Rua de Jorge Viterbo Ferreira 228, 4050-313 Porto, Portugal; 3 Interdisciplinary Center for Marine and Environmental Research (CIIMAR), University of Porto (U. Porto), Avenida General Norton de Matos, 4450-208 Matosinhos, Portugal

**Keywords:** Comet assay, Desiccation, DNA integrity, DNA oxidative damage, DNA strand breaks, 8-oxoG, Recalcitrant seeds, Single cell gel electrophoresis, Viability marker

## Abstract

**Background:**

Most plants encounter water stress at one or more different stages of their life cycle. The maintenance of genetic stability is the integral component of desiccation tolerance that defines the storage ability and long-term survival of seeds. Embryonic axes of desiccation-sensitive recalcitrant seeds of *Acer pseudoplatnus* L. were used to investigate the genotoxic effect of desiccation. Alkaline single-cell gel electrophoresis (comet assay) methodology was optimized and used to provide unique insights into the onset and repair of DNA strand breaks and 8-oxo-7,8-dihydroguanine (8-oxoG) formation during progressive steps of desiccation and rehydration.

**Results:**

The loss of DNA integrity and impairment of damage repair were significant predictors of the viability of embryonic axes. In contrast to the comet assay, automated electrophoresis failed to detect changes in DNA integrity resulting from desiccation. Notably, no significant correlation was observed between hydroxyl radical (^٠^OH) production and 8-oxoG formation, although the former is regarded to play a major role in guanine oxidation.

**Conclusions:**

The high-throughput comet assay represents a sensitive tool for monitoring discrete changes in DNA integrity and assessing the viability status in plant germplasm processed for long-term storage.

**Supplementary Information:**

The online version contains supplementary material available at 10.1186/s12870-021-03419-2.

## Background

Single-cell gel electrophoresis, also known as the comet assay, is a technique that is widely used to evaluate the formation and repair of DNA strand breaks and nucleobase modifications. The method is sensitive and cost-efficient, with proven diagnostic value in several areas of research, including biomonitoring, genotoxicity, carcinogenesis, and aging. Interest in this method has also attracted considerable attention from industry [1–6]. It has many widespread, potential applications including the study of terrestrial and aquatic ecosystems [4]. Since the alkaline comet assay was first developed for studying individual mammalian lymphocytes [7], it has been used in the study of multiple and diverse animal cells and tissues [8–15]. Any eukaryotic cell type that can be obtained as single cells, or as a nuclear suspension, appears to be amenable to comet assay analysis [16].

During single cell gel electrophoresis, supercoiled DNA, relaxed by breaks, is pulled toward the anode, and forms characteristic comets [17]. The relative amount (%) of DNA in the comet tail indicates the frequency of DNA breaks [16]. The comet assay can be performed under either neutral or alkaline conditions. While both versions provide evidence of double- and single-strand breaks, when performed under alkaline conditions, the comet assay can also detect apurinic or apyrimidinic sites (AP sites; alkali-labile sites, ALS) since it converts them into single-strand breaks, thus, increasing the sensitivity of the assay. ALS arise from a removal of damaged nucleobases, leaving the sugar backbone as intermediates in BER (base excision repair) or may arise spontaneously due to altered chemical stability [5, 17].

An essential step in the development of the comet assay for use in plant studies was the introduction of the mechanical extraction of cell nuclei to overcome problems caused by the presence of plant cell walls [18–20]. Many plant researchers have since developed their own protocols for the species under investigation as they can significantly differ in physical size, anatomy, physiology and genome size [5]. The comet assay has been primarily used to assess the level of stress imposed on a plant by environmental factors [21], although it has also been used in laboratory assessments of osmotic stress or the impact of irradiation and chemical compounds [22–26]. Most comet assays in plants have been performed on roots, leaves, or young needles [20, 21, 23, 24, 26, 27], although several studies have also demonstrated application of this method for the study of seed radicles at the protrusion phase [24, 28] and in the study of dry or imbibed seeds subjected to ionizing radiation or chemical treatments [22, 29–31].

Desiccation tolerance is a feature of seeds that enables their long-term survival under adverse conditions through processes that reduce the metabolic activity of cells and mitigate the mechanical stress imposed by cell shrinkage. These processes, common in orthodox seeds, allow seeds to tolerate water withdrawal below 10% of moisture content (MC). The cytoplasm solidifies at such a low level of MC, forming a glass, which drastically slows chemical reactions, enabling the preservation of entire seeds at low temperatures [32, 33]. In contrast, the excision of embryonic axes from hydrated, metabolically-active, recalcitrant seeds with limited or no desiccation tolerance is needed to preserve the genetic diversity present in such seeds during long-term storage in germplasm repositories. Recalcitrant embryonic axes must undergo partial desiccation and cryoprotection followed by storage in liquid nitrogen (LN) to ensure their viability and subsequent in vitro regeneration [34, 35]. Therefore, some degree of desiccation is still an essential step in the pre-storage procedure. Notably, the desiccation tolerance inherent in the seeds of different plant species is fundamental to the ability to preserve the viability of germplasm during long-term storage without detectable injury. Therefore, research on tissue sensitivity to desiccation is one of the most intensively explored topics in seed biology, seed storage research, and cryobiotechnology. Recalcitrant seeds of *Acer pseudoplatanus* L. were used in the present investigation. Sycamore seeds do not undergo maturation-drying while still attached to the maternal tree and should not be desiccated below 30% MC. They can only be stored under conventional conditions for 2–3 years at − 3 °C. For this reason, sycamore seeds have become a model system for desiccation studies of temperate, recalcitrant tree seeds [36–38].

The functional mechanisms responsible for desiccation tolerance involve LEA proteins, small heat shock proteins, non-reducing oligosaccharides, and antioxidants [39]. An increasing number of studies, however, have revealed that DNA damage and repair capacity are also major factors that determine germplasm viability [40]. Indeed, the loss of genomic integrity is one of the first stages of germplasm deterioration [30]. Understanding the mechanism of DNA damage and repair, and the development of a method to assess and monitor genome integrity, will improve the application and reliability of ex-situ preservation of plant genetic resources in gene banks.

The metabolism of all aerobic organisms, including plants, is based on an array of redox processes. Reactive oxygen species (ROS), as products of constitutive metabolism, are continuously generated in plant tissues as secondary messengers in regulatory events, but are also components of the initial response of plants to various biotic and abiotic stresses, including desiccation. When present in excess, however, ROS are one of the main causes of tissue damage [39, 41]. They are highly reactive molecules that can cause a wide range of cellular damage and a loss of function in macromolecules, including lipids, proteins and nucleic acids [39]. ROS have a high potential to induce DNA strand breaks (SB) that can be extremely deleterious and threaten genomic integrity [42]. In most cases, superoxide anion radicals (O_2_^٠─^) are the primary type of ROS produced, and can be readily converted to hydrogen peroxide (H_2_O_2_) and other peroxides involved in the Fenton reaction, yielding hydroxyl radicals (^**·**^OH), the most aggressive form of ROS [39, 43, 44]. The chemistry of ^**·**^OH with DNA is “messy” as it may form adducts to all four bases, and removes hydrogen atoms from 2′-deoxyribose, leading to strand scission, base release and/or oxidation, with guanine (G) being the most sensitive to oxidation. The most recent reports indicate that in model in vitro conditions, carbonate radicals (CO_3_^**٠**─^), rather than ^**·**^OH, are the major product of the Fenton reaction. CO_3_^**٠**─^specifically generates C8 or C5-oxidized G but does not cause direct DNA strand breaks, whereas, the activity of ^**·**^OH mainly results in sugar-phosphate oxidation products [43–47]. ROS formation in highly localized concentrations, however, may enable ^٠^OH to exert damage to DNA, including the formation of 8-oxo-7,8-dihydroguanine (8-oxoG) when oxidative stress is severe [43–45, 48].

In the present study, we wanted to determine how strong a desiccation stress was needed to represent a threat to DNA integrity. Therefore, we assessed the impact of progressive levels of desiccation on DNA strand breaks and oxidative damage to nucleobases (8-oxoG) in the embryos of *A. pseudoplatanus*. This required us to optimize the alkaline comet assay protocol to conduct this unique study, since earlier studies performed on seeds only utilized a neutral comet assay which is less sensitive than the alkaline comet assay [22, 29–31]. In addition, we also utilized an enzyme-modified comet assay to detect oxidized G, in which formamidopyrimidine DNA glycosylase (FPG) specifically recognizes and removes 8-oxoG [49]. The potential monitoring applications of the comet assay in germplasm storage facilities are also discussed.

## Results

### Viability of explants: TTC staining assay, survival, and regrowth

**Fig. 1:**
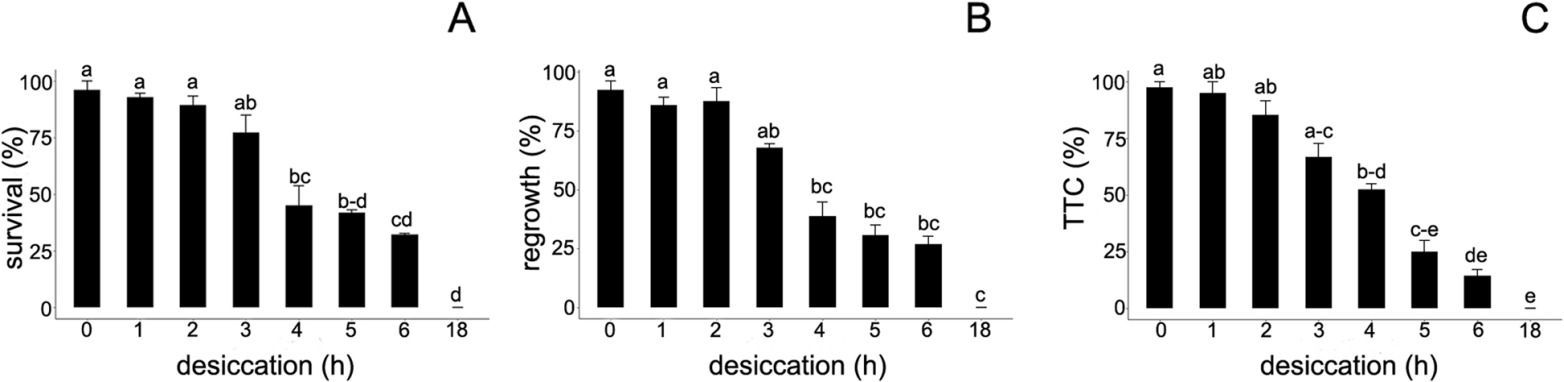
Effect of desiccation on *Acer pseudoplatanus* L. embryonic axes (**A**) survival (n = 5), (**B**) regrowth (n = 5) and (**C**) respiratory activity measured by TTC assay (n = 4). Values labelled with different letters are significantly different at p ≤ 0.05, according to Duncan test. Data represent mean ± SE

The impact of desiccation on embryonic axes was assessed as the survival of tissue and regrowth of seedlings in in vitro culture, as well as the level of metabolic activity of the tissues as measured with a 2,3,5-triphenyltetrazolium chloride (TTC) staining assay. The MC and water content (WC; g H_2_O^**.**^g^− 1^ dry weight) of embryonic axes are presented in Table 1. The highest percentage of survival (96%) of embryonic axes was observed in the control, non-desiccated samples (Fig. 1A). Gradual desiccation resulted in a decrease in survival, which became statistically significant after 4 h of desiccation (45.2%). An average of 32.1% of axes survived after 6 h of desiccation, while all samples were dead after 18 h of desiccation. Embryonic axes desiccated for up to 2 h had a high regrowth potential ranging between 92.3–85.9% (Fig. 1B). Further drying reduced this potential, with embryonic axes exhibiting 39.8% regrowth after 4 h and 27% after 6 h of desiccation. None of the embryonic axes developed a shoot after being exposed to 18 h of desiccation. A total of 97.5% of the control embryonic axes were viable according to the TTC staining assay (Fig. 1C). A statistically significant decrease (52.5%) in TTC staining was observed after exposure to 4 h of desiccation, followed by a decline to 14.3% after 6 h of desiccation. None of the embryonic axes exhibited TTC staining after exposure to 18 h of desiccation.

**Table 1:** Moisture content (MC) and water content (WC) of *Acer pseudoplatanus* L. embryonic axes after desiccation between 1 and 18 h and rehydration for 1 and 18 h, ±SE (*N* = 9)

Drying time (h)	t 0 h	t 1 h	t 18 h
	MC, % (WC, g^**− 1**^ dry weight)	MC, % (WC, g^**− 1**^ dry weight)	MC, % (WC, g^**− 1**^ dry weight)
	50.2 ± 0.08	62.5 ± 2.1	66.6 ± 0.06
0	(1.01 ± 0.03)	(1.68 ± 0.16)	(1.99 ± 0.05)
	19.3 ± 0.07	58.3 ± 1.9	71.8 ± 0.8
1	(0.24 ± 0.01)	(1.41 ± 0.07)	(2.55 ± 0.1)
	11.9 ± 0.08	54.2 ± 2.2	60.9 ± 4.6
4	(0.13 ± 0.01)	(1.19 ± 0.1)	(1.64 ± 0.35)
	9.2 ± 0.4	55.8 ± 0.09	60.4 ± 11.3
6	(0.10 ± 0.01)	(1.26 ± 0.05)	(2.53 ± 0.65)
	5.7 ± 1.2	64.5 ± 5.1	74.1 ± 2.5
18	(0.06 ± 0.01)	(1.89 ± 0.41	(2.91 ± 0.38)

### Optimization of the comet assay protocol: comparison of nuclei isolation buffers and the influence of lysis

**Fig. 2:**
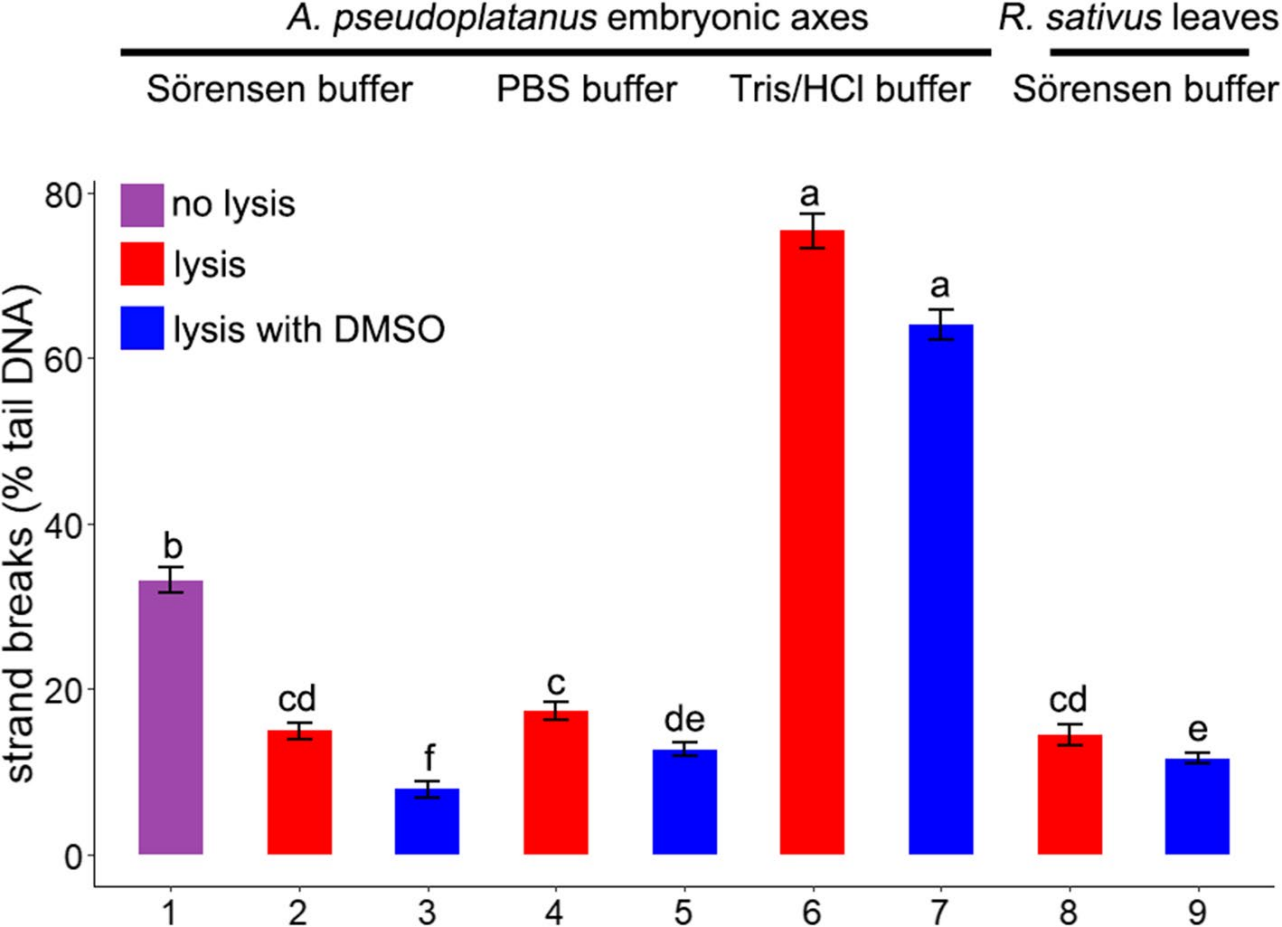
Influence of nuclei isolation buffer (supplemented with 0.5 mM Na_2_EDTA and 0.5% DMSO) and presence of lysis step on DNA strand breaks (% DNA in the tail). Nuclei were isolated from embryonic axes of *Acer pseudoplatanus* L. (1–7) and young leaves of *Raphanus sativus* L. (8–9). Values labelled with different letters are significantly different at *p* ≤ 0.05, according to Duncan test. Data represent mean ± SE, *n* = 3

The effect of different nuclei isolation buffers, as well as presence of DMSO, which functions as an antioxidant, was analyzed to determine the assay conditions that result in the lowest percentage of detected basal DNA strand breaks when using the alkaline version of the comet assay. The % of tail DNA in comets derived from control embryonic axes was analyzed for three variations of nuclei isolation buffers in the presence or absence of a lysis solution with and without DMSO (Fig. 2). The highest level of DNA damage was observed when nuclei were isolated in Tris-HCl buffer with or without 10% DMSO in the lysis solution (75.4 and 64%, respectively). In contrast, the lowest DNA damage was observed when Sörensen buffer was used followed by lysis with and without DMSO (14.9 and 7.8%, respectively). Samples prepared in a 1x PBS buffer in the presence or absence of DMSO also exhibited a low level of DNA damage (12.6 and 17.4%. respectively). Nuclei isolated from young leaves of *Raphanus sativus* L. using Sörensen buffer exhibited a % tail DNA similar to that obtained in nuclei isolated from embryonic axes of *A. pseudoplatanus*. Based on these results, subsequent assays utilized Sörensen buffer for the extraction of nuclei followed by lysis in a buffer supplemented with 10% DMSO.

### Validation of the comet assay protocol

**Fig. 3:**
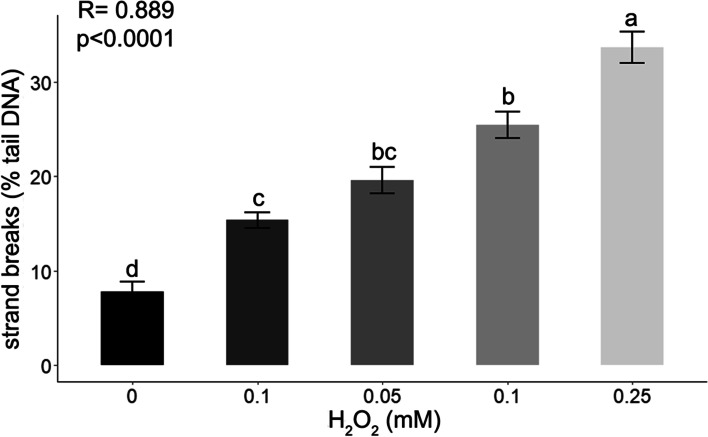
DNA strand breaks (% DNA in the tail) in nuclei isolated in Sörensen buffer from embryonic axes of *Acer pseudoplatanus* L. (MC of 50%) treated with H_2_O_2_ for 1 h at RT. Values labelled with different letters are significantly different at *p* ≤ 0.05, according to Duncan test. Data represent mean ± SE, *n* = 3

After optimization of the comet assay protocol, verification of the assay reliability was assessed using both a hydrogen peroxide (H_2_O_2_) treatment and the application of a tissue-aging treatment. The aim of these experiments was to determine if a broad range of DNA strand breaks (from several % up to 80–90% strand breaks) could be detected. Two approaches were evaluated: 1) Nuclei isolated from embryonic axes and embedded in agarose were subjected to different concentrations of H_2_O_2_ for 10 min on ice. Results indicated that a high level of DNA strand breaks (83.2 ± 8.8% SD) was detected at all of the H_2_O_2_ concentrations used (Supplementary file Fig. S1); 2) Embryonic axes were placed on a filter paper that had been soaked in H_2_O_2_ solutions ranging from 0.01–0.25 mM and left for 1 h on ice. The results indicated a significant dose response in the tested range of H_2_O_2_ concentrations (R = 0.889; *p* < 0.0001) (Fig. 3).

**Fig. 4:**
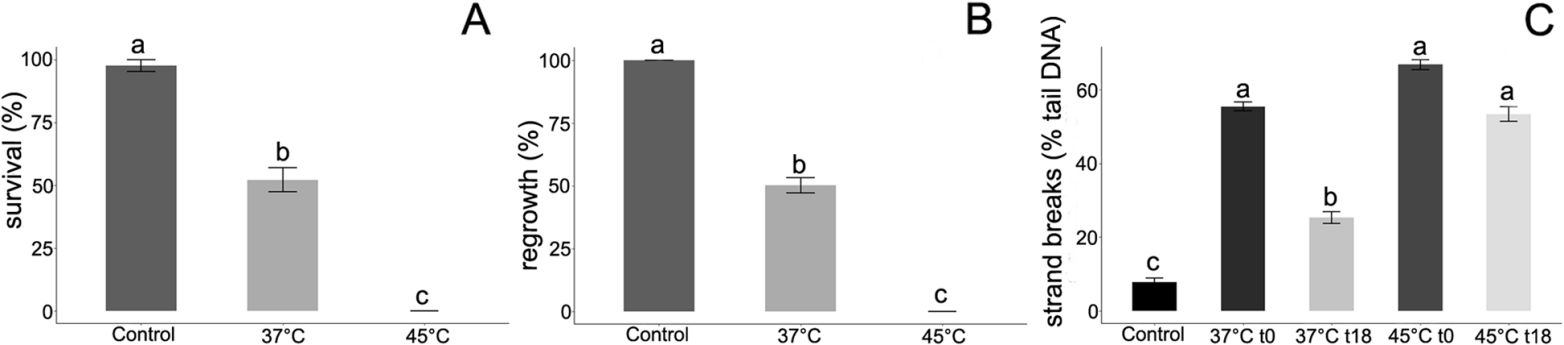
Embryonic axes of *Acer pseudoplatanus* L*.* (MC of 50%) were subjected to accelerating ageing conditions (storage at 37 and 45 °C; t0) and post-ageing recovery for 18 h at 4 °C (t18). **A** Survival (*n* = 5) and **B** regrowth (n = 5) were evaluated in in vitro cultures, **C** DNA strand breaks were evaluated using the alkaline comet assay (*n* = 3). Nuclei were isolated in Sörensen buffer supplemented with 0.5 mM Na_2_EDTA and 0.5% DMSO. Values labelled with different letters are significantly different at *p* ≤ 0.05, according to Duncan test. Data represent mean ± SE

Fresh embryonic axes were also subjected to accelerated aging by placing them at a high MC (50%) and temperatures of either 37 °C or 45 °C for 3 days to determine if comets with large tails could be assessed and if the process of DNA repair could be observed. The level of DNA strand breaks was measured immediately after each treatment condition, as well as after 18 h of recovery on water-soaked filter paper at 4 °C. Both temperatures resulted in a significant increase in DNA strand breaks, reaching the levels of 55.5 and 66.9% in the 37 °C and 45 °C treatments, respectively. Notably, a statistically significant decrease in the % of DNA in comet tails after 18 h of recovery suggesting DNA repair was only observed in embryonic axes kept at 37 °C (Fig. 4C). Even after the repair period, however, the % of DNA in the tail was higher (25.3%) than it was in the control samples. Importantly, the axes subjected to 37 °C exhibited ~ 50% survival and regrowth, while all of the embryonic axes incubated at 45 °C were dead when the viability of treated embryonic axes was assessed (Fig. 4A, B).

### Desiccation of embryonic axes results in DNA damage and impairment of DNA repair

**Fig. 5:**
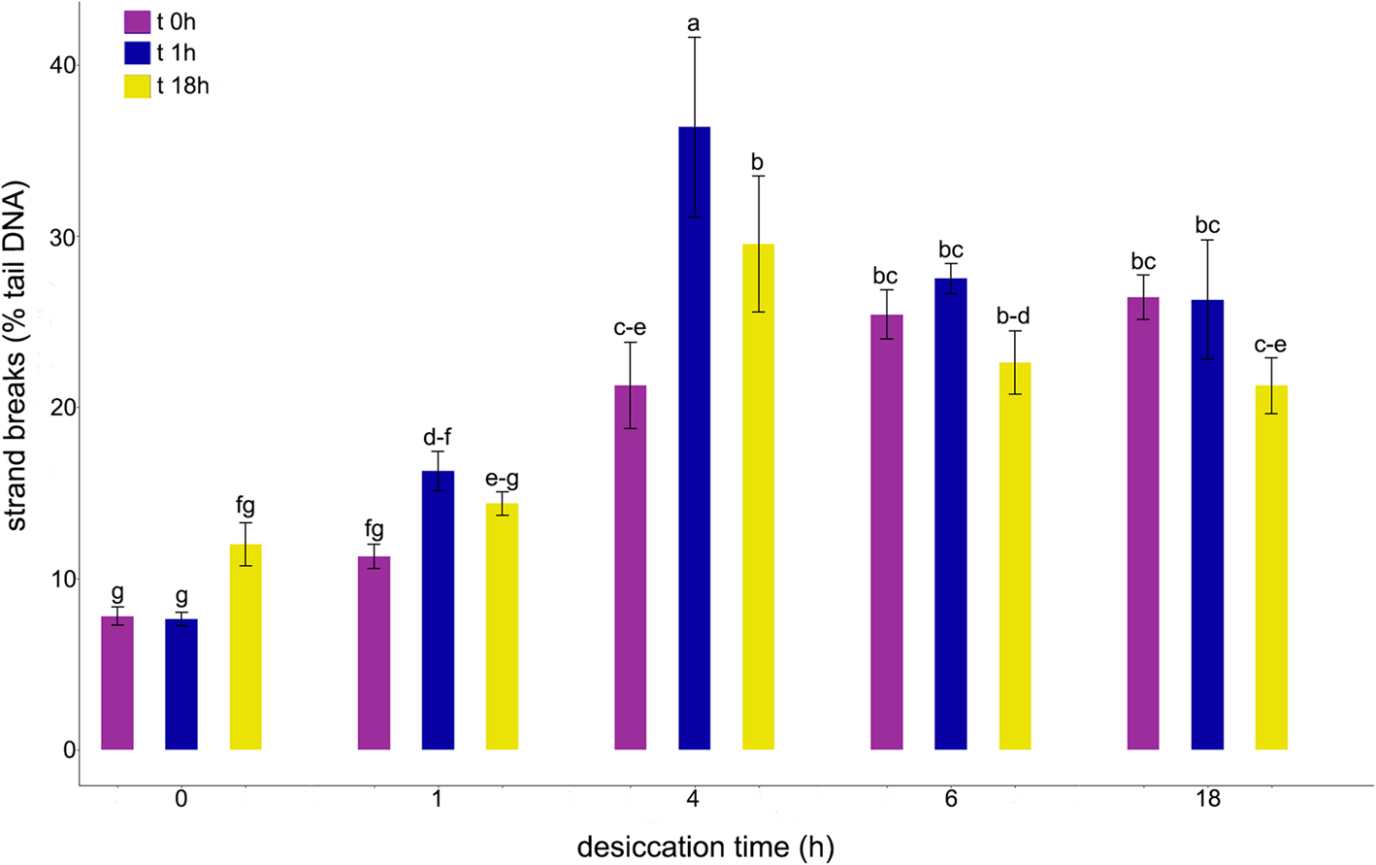
Influence of gradual desiccation at ambient temperature (t 0 h) and subsequent rehydration for 1 (t 1 h) and 18 h (t 18 h) on DNA strand breaks (% DNA in the tail) representing single, double strand breaks and alkali-labile sites measured with the alkaline comet assay. Nuclei were isolated in Sörensen buffer supplemented with 0.5 mM Na_2_EDTA and 0.5% DMSO from embryonic axes of *Acer pseudoplatanus* L. Values labelled with different letters are significantly different at *p* ≤ 0.05, according to Duncan test. Data represent mean ± SE, *n* = 3

**Fig. 6:**
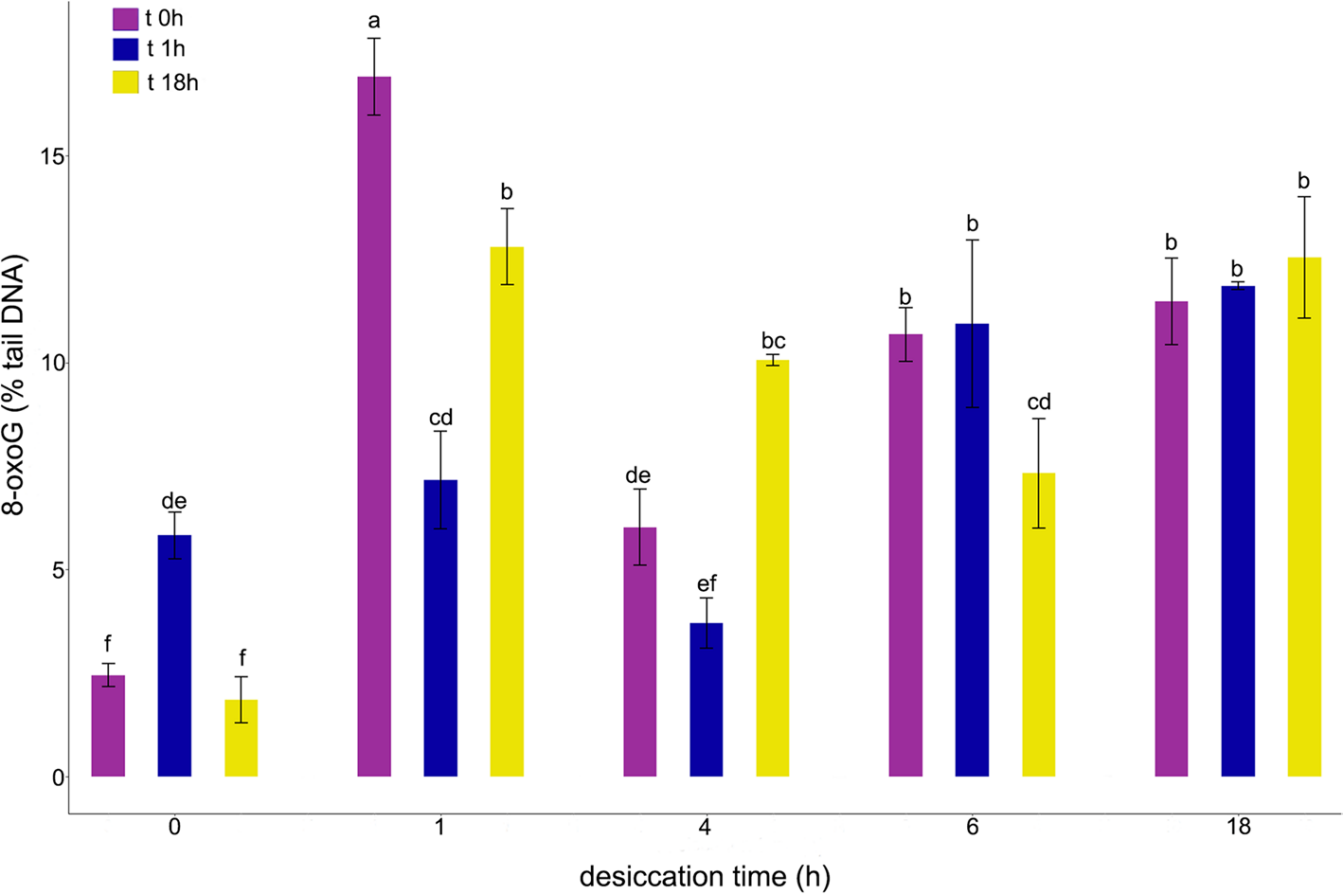
Influence of gradual desiccation at ambient temperature (t 0 h) and subsequent rehydration for 1 (t 1 h) and 18 h (t 18 h) on 8-oxoG level (% DNA in the tail) measured with FPG enzyme-modified alkaline comet assay. Nuclei were isolated from embryonic axes of *Acer pseudoplatanus* L. in Sörensen buffer supplemented with 0.5 mM Na_2_EDTA and 0.5% DMSO. Values labelled with different letters are significantly different at *p* ≤ 0.05, according to Duncan test. Data represent mean ± SE, *n* = 3

The effect of desiccation on the formation of DNA strand breaks and 8-oxoG lesions was measured immediately after different periods of desiccation and after 1 h and 18 h of rehydration (Figs. 5 and 6). The level of basal DNA strand breaks in the control (not desiccated) axes was 7.8% (Fig. 5). The percentage of strand breaks increased to 11.3 and 21.1% after 1 h and 4 h of desiccation, respectively. Furthermore, after 1 h of rehydration, % tail DNA increased in embryonic axes desiccated for 1 h and 4 h (16.3 and 36.4%, respectively). After further rehydration for 18 h, however, the level of DNA strand breaks decreased in embryonic axes desiccated for 1 h and 4 h to 14.4 and 29%, respectively. Notably the percentage of strand breaks only returned to control levels in axes desiccated for 1 h. No significant change in DNA breaks was observed after 1 h or 18 h of rehydration in samples of embryonic axes desiccated for 6 h and 18 h (25.4 and 26.5%, respectively).

The percentage of 8-oxoG lesions in control embryonic axes was estimated to be 2.45% (Fig. 6). The % tail DNA in comets of non-lysed nuclei was estimated to be 10.1 ± 2.81% SD. The 1 h desiccation treatment induced the highest level of 8-oxoG (16.9%), followed by a decline (7.17%) and then a further increase to 12.8% after 18 h of rehydration. The 4 h desiccation treatment resulted in 6.03% of DNA in the tail followed by a decrease to 3.7% after 1 h of rehydration and a subsequent increase to 10.1% after 18 h of rehydration. The observed changes were statistically significant. No difference in the % tail was observed in samples assayed directly after 6 h of desiccation and after 1 h of rehydration (10.7, 10.9%, respectively), however the % tail decreased to 7.3% after 18 h of rehydration. No significant change between samples desiccated for 18 h was observed (11.5–12.5% of tail DNA).

### Measurement of reactive oxygen species

**Fig. 7:**
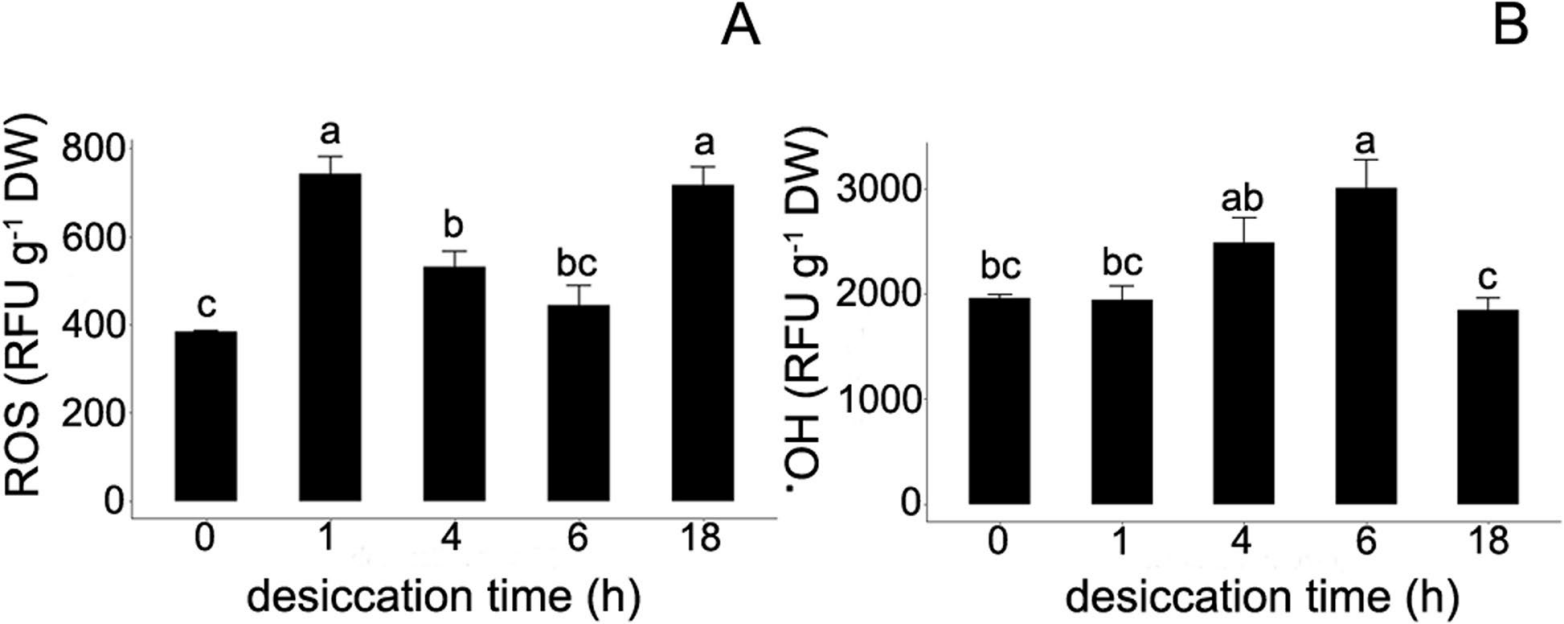
Influence of gradual desiccation on ROS (A) and ^٠^OH (B) level in embryonic axes of *Acer pseudoplatanus* L. Values labelled with different letters are significantly different at *p* ≤ 0.05, according to Duncan test. Data represent mean ± SE, *n* = 4

The impact of gradual desiccation on ROS and ^٠^OH accumulation were separately measured. ROS levels were determined using the H_2_DCFDA assay, which is based on fluorescent measurements of 2′,7′-dichlorofluorescein (DCF). Desiccation for 1 h almost doubled the fluorescence signal in embryonic axes, relative to the non-desiccated, control samples (385 vs. 741 RFU g^− 1^ DW; Fig. 7A). Further desiccation, however, resulted in decreased ROS levels and were comparable to control samples, although 18 h of desiccation induced an increase in fluorescence up to 718 RFU g^− 1^ DW. The highest level of ^٠^OH was detected after 6 h of desiccation (3003 RFU g^− 1^ DW; Fig. 7B). The level of ^٠^OH ranged from 1944 after 1 h of desiccation to 1842 RFU g^− 1^ DW after 18 h of desiccation.

### DNA fragmentation

**Fig. 8:**
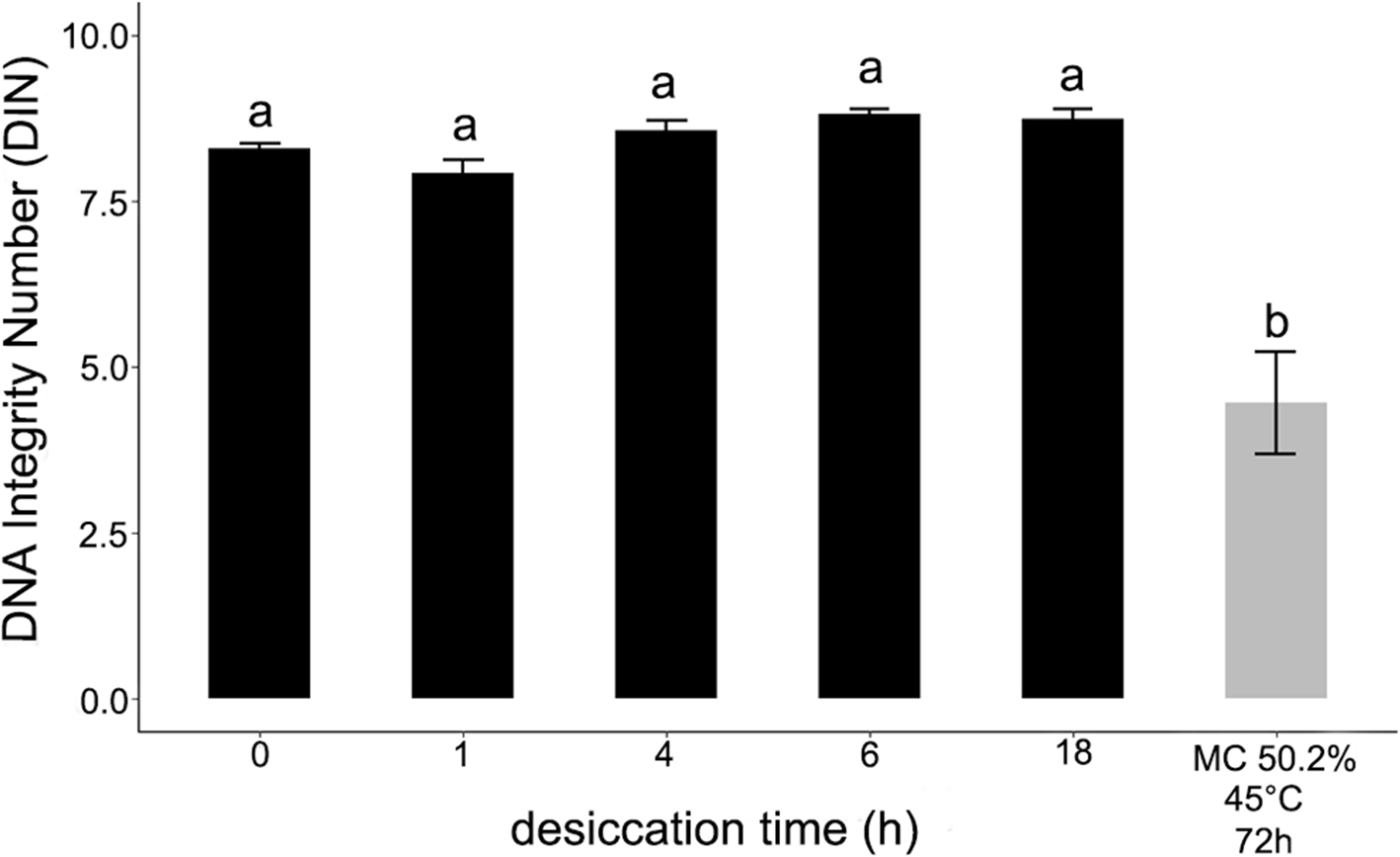
Influence of gradual desiccation and accelerated aging on DNA isolated from embryonic axes of *Acer pseudoplatanus* L. DNA fragmentation was analyzed by automated electrophoresis in a range from 200 to > 60,000 bp. For accelerate aging embryonic axes were kept for 72 h at 45 °C. Values labelled with the same letters are not significantly different at *p* ≤ 0.05, according to Duncan test. Data represent mean ± SE, *n* = 3

The ability to detect desiccation-induced DNA fragmentation by automated electrophoresis was also investigated. Desiccation of embryonic axes did not result in detectable DNA fragmentation in the range of 200–60,000 bp or 35–1000 bp (Fig. 8; Supplementary file Fig. S2). Analysis of the higher bp range revealed no statistical difference between samples with DNA integrity (DIN) values ranging from 7.92 to 8.82. When embryonic axes subjected to an accelerating aging treatment were evaluated as a positive control, the DIN value decreased to 4.45. When DNA fragmentation in the smaller bp range was analyzed, an additional peak at around 250 bp, suggesting DNA degradation, was observed only in the path corresponding to DNA isolated from aged embryonic axes.

### Correlation and principal component analyses

**Fig. 9:**
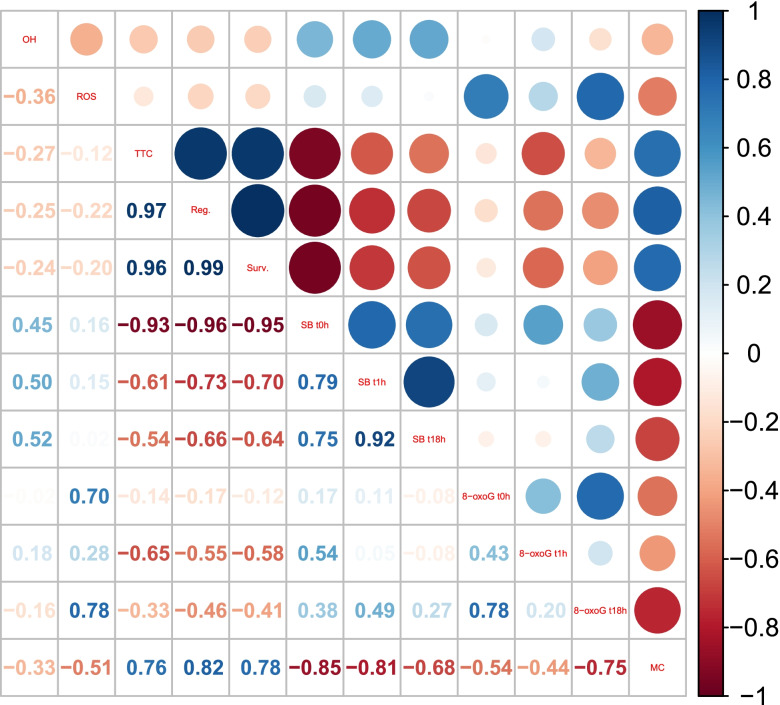
The Spearman correlation coefficient between the means of oxidative stress measurements (^٠^OH, ROS), viability measurements (TTC, regrowth, survival), DNA strand breaks measured directly after desiccation (SB t0h), after 1 h of rehydration (SB t1h) or after 18 h of rehydration (SB t18h), and the amount of detected 8-oxo G measured directly after drying (8-oxoG t0h), after 1 h of rehydration (8-oxoG t1h) or after 18 h of rehydration (8-oxoG t18h). The size of the circles represents the level of correlation (r), bigger circles indicate that a given trait correlates at higher level. Blue color indicates positive correlations and red indicates negative correlations

A very strong negative correlation was observed between the viability of embryonic axes and the presence of DNA strand breaks. The levels of 8-oxoG and ROS were also negatively correlated with viability (Fig. 9). MC was significantly, negatively correlated with DNA strand breaks. Notably, no significant correlation was observed between the MC level of embryonic axes and the level of hydroxyl radicals. The strongest negative correlation between measurements of 8-oxoG and the viability of embryonic axes was observed after 1 h of rehydration (Fig. 9).

**Fig. 10:**
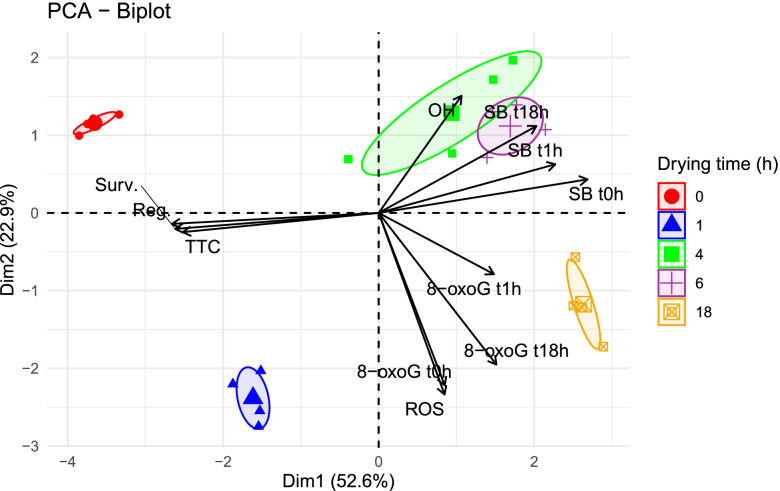
Result of principal component analysis (PCA) applied on the correlations of survival (Surv.), regrowth (Reg.), metabolic activity (TTC), ROS level (ROS), hydroxyl radical (^٠^OH), relative amount (%) of DNA strand breaks in desiccated embryonic axes (SB t0h), after 1 h of rehydration (SB t1h), and after 18 h of rehydration (SB t18h), and relative amount (%) of 8-oxoG measured in desiccated embryonic axes (8-oxoG t0h), after 1 h of rehydration (8-oxoG t1h), and after 18 h of rehydration (8-oxoG t18h). Ellipse confidence level = 0.95

The PCA results indicated strong negative relationships between the viability of embryonic axes (both desiccated and rehydrated) and DNA strand breaks and the level of 8-oxoG after 1 h of rehydration (Fig. 10). The strongest negative relationship was detected between in vitro regrowth of embryonic axes (coordinates − 0.9523) and the level of DNA strand breaks measured in desiccated embryonic axis (coordinates 0.9479). This negative relationship was reflected in the opposite coordinates of these two groups and the high loading of these variables in principal component 1 (Prin1), (Supplementary file Tables 1 and 2). ROS and 8-oxoG levels in desiccated embryonic axes and the 8-oxoG level in embryonic axes rehydrated for 18 h had a major impact on Prin2, and these three variables exhibited a strong positive relationship. The level of ^٠^OH also had a significant influence on Prin2, although it exhibited a negative relationship with ROS levels, 8-oxoG in desiccated embryonic axes, and with the level of 8-oxoG in axes rehydrated for 18 h. The first two principal components accounted for 55.5 and 22.7% of the observed variance, respectively (totaling 78.2%). Based on Prin1 and Prin2, the embryonic axes clustered into five groups corresponding to their MC.

## Discussion

Induced desiccation of germplasm is used in gene banks before seeds or explants are placed in storage at low temperature. The methodology is based on a combination of drying and cooling to prevent the formation of intracellular ice crystals. In contrast to orthodox seeds, recalcitrant seeds do not survive the level of drying needed to solidify the cytoplasm in their cells. Excised embryos of recalcitrant seeds, however, can nevertheless be stored at cryogenic temperatures after partial dehydration [50]. Therefore, it is important to investigate the impact of desiccation on the genetic stability of preserved material. Such studies provide important knowledge about processes underlying desiccation sensitivity and may serve as a molecular assay of the storability of recalcitrant germplasm.

Although the comet assay was primarily developed for animal cells, its application to plant tissues has significantly grown after the introduction of some modifications. To the best of our knowledge, the present study is the first to investigate the use of the comet assay technique to analyze the impact of desiccation on seed DNA damage and repair. The comet assay protocol was optimized and validated in our study as a tool for the prediction of the viability of embryonic axes based on the changes in DNA integrity. Embryonic axes excised from recalcitrant (desiccation-sensitive) seeds were used as a model system. The use of embryonic axes enabled us to assess DNA damage and repair in a uniform tissue, in contrast to using the entire embryo where the embryonic axis and cotyledons often differ in their response to desiccation [38].

Mechanical isolation of nuclei in plant tissues was previously reported to have been conducted in PBS [22, 31], Sörensen [20], and Tris-HCl buffers [21, 23, 26, 29], which differ in osmolarity and ion concentrations. Other studies used these buffers supplemented with Na_2_EDTA and with or without DMSO [24, 28, 51]. There are also variations in the composition of the lysis solution used to disrupt the nuclear membrane and protein components [23, 27, 30, 43, 44]. Several reports concluded, however, that the lysis step is redundant [20, 21, 52]. In the current study, three nuclei isolation buffers supplemented with Na_2_EDTA and DMSO were evaluated in conjunction with two variants of lysis solution (Fig. 2). The lowest basal level of strand breaks in non-desiccated, control axes was observed in nuclei isolated in Sörensen buffer lysed in a solution supplemented with 10% DMSO. Notably, the presence of DMSO in the lysis solution decreased % DNA observed in the comet tail in all of the tested lysis variants. Importantly, however, when the comet assay was performed on nuclei isolated in Sörensen buffer from young leaves, the presence of DMSO in the lysis buffer also affected the % of DNA in the tail, but to a lesser extent (2.8% vs. 7.1%). Thus, we demonstrated that the optimized protocol we developed is also suitable for tissues other than embryonic axes. Although a protective role of DMSO as an ^٠^OH scavenger is suggested [53], our results may also suggest that a difference in redox-potential exists between different tissues. In fact, the comet assay has been proposed as a method to assess the antioxidant status of cells [16]. Notably, the results of our study are contradictory to a previously published report [21] that determined that the lysis step did not affect the comet assay results to a measurable extent, at least for nuclei isolated from leaves and roots. In our study, nuclei not subjected to lysis had a baseline of 30% tail DNA which was higher than levels previously reported for nuclei isolated from leaves (12.9 and 5.1%) [21, 52]. Since comets from untreated nuclei should generally have a background level of breaks corresponding to ≤5% of tail DNA, a higher background (> 10%) under normal conditions suggests poor comet assay parameters that require a modification of the assay conditions [5]. The lysis step appears to be necessary to limit the formation of unspecific DNA breaks possibly by the dismantling of cellular components that generate ROS (mitochondria, peroxisomes, chloroplasts) and scavenging of ^٠^OH by DMSO. Differences in antioxidant status, total composition of chemical elements, and metabolic activity between tissues may also explain the discrepancy between studies in the required use of a lysis step. This aspect, however, needs further investigation.

It was striking that the level of DNA damage was the highest when nuclei were isolated in Tris-HCl buffer (Fig. 2), as this buffer has been previously used for the isolation of nuclei from roots, radicles, leaves, and needles [21, 23, 26, 29, 54] but not from seeds. When Tris-HCl/Na_2_EDTA was used to isolate nuclei from radicles, the basal level of DNA strand breaks in the non-treated control [24], recalculated into a pseudo-percentage score [55], ranged between 15 and 23%. The differences may result from the variation in the inherent features of the tested samples related to differences in chemical or antioxidant composition, or from differences in the effect of the ionic strength of the buffer on chromatin condensation and its accessibility to be damaged by ROS [56]. Phosphate buffers are known to maintain pH at low temperature and sequester divalent cations required for DNase activity, which could affect the level of strand breaks. Nevertheless, based on our results, we recommend that nuclei should be isolated in Sörensen buffer and subjected to lysis, as in these conditions the basal level of DNA strand breaks was the lowest.

After the optimization of the nuclei isolation, it was essential to verify the reliability of the assay. For that purpose, a dose-dependent damage response to increasing concentrations of H_2_O_2_ was analyzed (Fig. 3). Although the concentration of the damage inductor was 4.5 x higher than previously used [21] and exposure of embryonic axes for 1 h to H_2_O_2_ resulted in a significant dose-response, the maximal damage observed was ca. 34%. This comparatively low level of DNA strand breaks may have been due to the method of treatment that was used. While H_2_O_2_ produces strand breaks, the level of breaks varies widely from cell type to cell type and even from cell to cell, presumably due to the presence of differing levels of antioxidants [16]. Therefore, to further investigate the reliability of our assay and potential ability to detect a higher % of tail DNA and DNA repair activity, embryonic axes were subjected to accelerated-ageing conditions, represented by a combination of high moisture content and elevated temperature (Fig. 4). This treatment is known to initiate germplasm decay processes [57]. The effect of the accelerated-aging treatment on DNA integrity has been previously measured using the neutral comet assay in orthodox rice and common bean seeds [31]. The current results demonstrated that storage of germplasm at a high temperature caused a significant increase in DNA strand breaks (up to 56–67%). DNA repair, however, was only observed in samples stored at 37 °C. Storage at 45 °C resulted in cell death, which was manifested as a lack of regenerated tissues in in vitro culture and not a significant decrease in % tail DNA. The results on H_2_O_2_ dose-response and accelerated-aging conditions confirmed that the optimized comet assay could be used to monitor DNA damage and repair in stored germplasm and that a lack of DNA repair is associated with the decline in tissue viability observed in subsequent in vitro culture of stored materials.

In the current study, desiccation steps were adjusted to induce a gradual decrease in viability markers ranging from no significant decline after 1 h to a complete loss of viability. Further, the tolerance of embryonic axes to the applied desiccation steps was evaluated by monitoring DNA damage and subsequent repair upon rehydration, based on the assumption that if DNA repair capacity is preserved, loss of DNA integrity should not be an impediment to desiccation survival [58]. It has been previously reported that the first event in seed imbibition in water is the hydration of the cytoplasm. Then, as the uptake of water proceeds, metabolic activity is activated. Hydrated cells have the potential for rapid DNA repair when DNA repair enzymes begin to function. For example, DNA single-strand breaks in embryos of *Avena fatua* L. (orthodox) were repaired within 1.5 h [58, 59]. Our results indicate that there is an increase in DNA strand breaks after 1 h of rehydration, followed by a decrease, when embryonic axes are desiccated for 1 or 4 h (Fig. 5). When the % tail DNA in comets derived from embryonic axes desiccated for longer times was analyzed, no significant changes in response to rehydration were observed. The percentage of tail DNA after 18 h of rehydration differed significantly from the non-treated control except in embryonic axes desiccated for 1 h. Analysis of the viability results indicated that no significant change in viability were observed, as measured by the three indicated parameters, only in the embryonic axes desiccated for 1 h followed by 18 h rehydration. The significant negative correlation between viability and the alkaline comet assay results indicates that the monitoring of the formation and repair of strand breaks may serve as a predictive marker for the viability of embryonic axes. Notably, the strongest correlation with high statistical significance was observed for the % of tail DNA measured directly after desiccation (Fig. 9).

As the excision and desiccation of embryonic axes imposes oxidative stress, the level of 8-oxoG in DNA was assessed using an enzyme-modified comet assay (Fig. 6). The assay used Fpg enzyme, which primarily exhibits a substrate preference for oxidized purines, including 8-oxoG and 2,6-diamino-4-hydroxy-5-formamidopyrimidine (FaPyG). The enzyme detects and removes damaged bases, leaving abasic sites, which are converted to strand breaks by the associated AP-lysase activity of the enzyme [49]. At least 5 min of lysis was reported to be needed to allow the enzyme to access the DNA [60]. Importantly, in our results, the data on the relative amount of 8-oxoG reflected an opposite trend from the changes in the % tail of DNA analyzed by the alkaline comet assay. The increase in detected DNA strand breaks measured after 1 h of rehydration corresponded to a decrease in 8-oxoG level in embryonic axes desiccated for 1 and 4 h. The highest level of oxidized G, which was observed after 1 h of desiccation, may have been related to the high change in MC (30%) and the doubling of the level of ROS (Fig. 6A), although the amount of damage decreased after 1 h of rehydration. Notably, 18 h of rehydration imposed an obvious stress on tissues, which was reflected in an increase in 8-oxoG. However, it is important to note that this stress was only significantly negatively correlated with regrowth (Fig. 9). The high negative correlation between 8-oxoG levels and viability was observed for all of the viability markers after 1 h of rehydration, indicating that a higher level of 8-oxoG is present in tissues that have lower viability. Notably, rehydration-related changes in 8-oxoG were non-significant in embryonic axes desiccated for a longer time (6 h and 18 h), suggesting the failure of the DNA repair system, which is considered to be severely inhibited or inactivated by water loss [58].

Oxidized bases in DNA are usually removed in the base excision repair pathway (BER), which is active throughout the cell cycle. On the other hand, sugar oxidation products of 2’deoxyguanosine serve as substrates of nucleotide excision repair (NER) or single-strand break repair. During the BER and NER processes, DNA gaps are present due to damaged nucleotides or DNA fragment excision [40, 61]. Consequently, the increase in % tail DNA observed after 1 h in the alkaline comet assay may result from either or both DNA damage and the activity of repair pathways, which is in agreement with the observed decreasing levels of 8-oxoG. The observed changes in strand breaks and 8-oxoG level support the premise that if a highly efficient DNA repair can be maintained, fragmentation of DNA or other damage may not represent a serious impediment to post-desiccation regeneration. Notably, the lowest values for the measured viability parameters were observed in embryonic axes with impaired DNA repair. The impairment of DNA damage repair observed in rehydrated tissues may be attributed to lowered enzyme activity or enzyme inactivation caused by desiccation [32]. Our measurements of H_2_DCFDA, however, indicated a second peak in ROS levels after 18 h of desiccation. It is important to note that in addition to the major factors causing H_2_DCFDA oxidation, such as H_2_O_2_, cytochrome c and peroxidases in the presence of heme-containing compounds, lipid peroxides are also capable of generating DCF fluorescence [62, 63]. Therefore, the increase in the fluorescence signal may reflect membrane damage that has already been previously reported in desiccated *Acer pseudoplatanus* seeds [64]. This increase in oxidative potential, however, did not result in a significant increase in DNA strand breaks or 8-oxoG levels. This may potentially be attributed to the impact of desiccation-induced changes in DNA conformation resulting from the withdrawal of water molecules and the protective role of DNA-associated proteins [48, 50, 58, 59].

In the present study, the relationship between oxidative stress and oxidative DNA lesions was investigated. Results indicated that ^·^OH was not significantly correlated with the presence of 8-oxoG (Fig. 9), but rather with DNA strand breaks. Interestingly, no correlation was observed between ROS and ^**·**^OH measurements and the results of the viability assay (Fig. 9). This is in agreement with a previous report [41] that demonstrated a lack of relationship between different ROS (^٠─^O_2_), antioxidant capacity, and recalcitrant explant viability. It appears that biomarkers, such as ROS, are useful in describing the nature of desiccation stress, but may not be helpful as direct indicators of the actual viability of tissues.

The results obtained on DNA strand breaks present in desiccated embryonic axes were compared with results on DNA fragmentation derived from the use of automated electrophoresis conducted in the range 200–60,000 bp and 35–1000 bp. In previous research based on the electrophoresis of extracted genomic DNA in an alkaline agarose gel, a progressive loss of DNA integrity and increasing levels of random-sized small-molecular-weight fragments was observed in dry-stored aged orthodox seeds [65, 66]. In the present study, no statistical difference was observed between DNA extracted from control and desiccated embryonic axes, although a degradation was noted for DNA extracted from embryonic axes at 50% of MC subjected to accelerating-aging when analyzed in the range of 200 bp - > 60 kbp and 35–1000 bp (Fig. 8; Supplementary file Fig. S2). DNA integrity in aged seeds has previously been reported to be compromised, and DNA damage in the form of strand breaks and point mutations were correlated with lower seed germination and strand-break detection in plantlets [40, 58, 67]. Our results clearly demonstrated, for the first time, that the optimized comet assay protocol is more sensitive in the detection of DNA damage than automated electrophoresis and should be the method of choice for detecting viability-related changes in DNA integrity. Since the dynamic range of the comet assay is limited to a few hundred to several thousand breaks, it is misleading to interpret its results as DNA fragmentation as even at the maximum saturation level only a few thousand lesions per cell can be detected [5] which under physiological conditions are generally reparable [16]. Therefore, the modified comet assay can be used to detect fine, discrete levels of DNA damage and assess DNA repair efficiency.

## Conclusions

Both types of DNA damage (8-oxoG and DNA strand breaks) and their repair pathways contribute to mutagenic changes, genome rearrangements and instability, and are, thus, undesirable when conserving genetic resources. The present results support the premise that maintenance of DNA integrity is critical for successful germplasm preservation. The optimized comet assay developed in our study has demonstrated, for the first time, the formation of DNA strand breaks and 8-oxoG imposed by gradual desiccation stress and their repair in rehydrated tissues. Based on PCA analysis (Fig. 10), it is readily apparent that embryonic axes can be separated into five groups based on the measured parameters, which provides the ability to distinguish between control embryonic axes and axes subjected to desiccation and declining in viability. We propose that our optimized, high-throughput alkaline comet assay, combined with the FPG enzyme assay, represents an effective tool for monitoring the DNA integrity of stored germplasm resources and predicting its viability. Importantly, our optimized protocol also provides the ability to monitor the DNA repair process. The ability to monitor DNA damage, as well as understanding its sources and the presence of repair systems, is essential to understanding the desiccation tolerance of seeds and the development of appropriate techniques for long-term storage.

## Methods

### Plant material and treatments

Samaras of *Acer pseudoplatanus* L. (sycamore) were collected from trees in the provenances situated in Olsztyn (53.7553° N, 20.4561° E), in northern-eastern Poland. Dr. B. Plitta-Michalak and Dr. M. Michalak undertook the formal identification of the samples. This research examines a native, not endangered plant species and collection of samples for scientific purposes was permitted by local legislation. The voucher specimen has not been deposited in any publicly available herbarium. After harvest, the samaras were surface dried at room temperature and 35% RH on sheets of paper and subsequently stored at 3 °C in tightly sealed, plastic containers. Embryonic axes were excised from the embryos no later than 8 weeks after collection. All chemicals and materials were purchased from Merck (Darmstadt, Germany), unless otherwise indicated.

For the desiccation experiment, embryonic axes were placed on a filter paper over activated silica gel (60 g) in a closed Petri dish (120 mm diameter) which was sealed with parafilm. The duration of the desiccation protocol ranged from 1 to 18 h. The moisture content (MC) of embryonic axes was assessed by drying each axis at 103 °C ± 2 °C for 24 h and was calculated on a fresh weight basis as previously described [38, 68]. For rehydration, embryonic axes were placed on a paper filter soaked with 5 mL water in a 60 mm glass Petri dish sealed with parafilm and kept at 4 °C for 18 h. For the accelerated-ageing experiment (storage at high temperature and high MC), fresh embryonic axes at MC of 50% were kept in 2 mL tubes at either 37 °C or 45 °C for 3 days.

### Viability assessment

A tetrazolium chloride (TTC) assay was used based on the International Rules for Seed Testing [69]. Embryonic axes isolated from non-stratified seeds were soaked in a 1% solution of 2,3,5-triphenyltetrazolium chloride in 50 mM potassium phosphate buffer (pH 7.5) and incubated in the dark at 30 °C for 24 h. Four biological replicates comprising ten embryonic axes in each replicate were utilized in the TTC assay. Embryonic axes that remained green (unstained) were considered dead, while those stained pink to red were classified as alive and metabolically active.

In vitro survival and regrowth assays were conducted using five biological replicates, comprised of 10–15 embryonic axes in each replicate, that were isolated from non-stratified seeds. After desiccation, axes were kept for 18 h on a wet filter paper at 4 °C, to avoid excessive bleach absorption by tissues and subsequently surface-sterilized in 10% commercial bleach (≤5% NaClO and ≤ 1% NaOH) for 5 min followed by four rinses in sterile, distilled water. Control embryonic axes were treated identically to the axes used in the experiments. The embryonic axes were subsequently cultured on MS medium [38, 70] containing sucrose (30 g l^− 1^), 0.8 mg l^− 1^ 6-benzylaminopurine (BAP;) and agar (7.0 g l^− 1^) for solidification. The pH of the medium was adjusted to 5.7 prior to autoclaving. Embryonic axes were cultured at 19 °C with a 16 h/8 h, light/dark photoperiod and a light intensity of 77 μmol m^− 2^ s^− 1^.

The survival of embryonic axes was assessed after 4 weeks of in vitro culture. Axes were considered viable if they displayed evidence of shoot and root, shoot only, root only development, or had increased in size during the culture period. Regrowth of embryonic axes was assessed after 8 weeks and only counted as regenerated if the embryonic axis had developed either a shoot or a shoot with a root.

### ROS detection

ROS levels were quantified in control and desiccated embryonic axes using the fluorogenic dye, 2′,7′-dichlorodihydrofluorescein diacetate (H_2_DCFDA; Invitrogen, Waltham, MA, USA). The dye primarily detects hydrogen peroxide but recent evidence has shown that other ROS, such as hydroxyl radical, hydroperoxides, and peroxynitrite, can also oxidize H_2_DCF to a fluorescent product, however, with greatly reduced sensitivity relative to H_2_O_2_ [62, 63]. Embryonic axes were incubated for 15 min in 1 mL of 10 μM H_2_DCFDA in darkness on a shaker at 150 rpm. The supernatant was subsequently clarified by a short centrifugation. Fluorescence was measured at an excitation wavelength of 492 nm and an emission wavelength of 525 nm using an Infinite M200 PRO plate reader (Tecan, Männedorf, Switzerland). A sample containing only H_2_DCFDA was used as a negative control. Four replicates of five embryonic axes in each replicate were analyzed in each desiccation treatment. Results are expressed as relative fluorescence units per gram of dry weight (RFU g^− 1^ DW).

Four replicates, which were comprised of five embryonic axes in each replicate, were used for the estimation of hydroxyl radical (^٠^OH) levels. The axes were immersed in 1 mL of 5 mM sodium benzoate in 20 mM potassium phosphate buffer (pH 6.0) and incubated on a rotary shaker at 150 rpm for 3 h at room temperature in darkness. The supernatant was then clarified by a short centrifugation [71]. Fluorescence was measured at an excitation wavelength of 305 nm and an emission wavelength of 407 nm using an Infinite M200 PRO (Tecan, Männedorf, Switzerland) plate reader. A sample containing only sodium benzoate was used as a negative control. Results are expressed as relative fluorescence units per gram of dry weight (RFU g^− 1^ DW).

### The comet assay

The entire procedure was performed under subdued light and the slides and nuclei suspension were kept in darkness to avoid light-induced damage. The comet assay was conducted using a high-throughput Compac-50 system that includes a horizontal electrophoresis tank for 50 slides and a CS-300 V Power Pack power supply (Cleaver Scientific, Warwickshire, UK).

Embryonic axes excised from seeds or 5 mm × 10 mm fragments of leaves of *Raphanus sativus* L. plantlets were placed in a 60 mm glass Petri dish, kept on ice, and vigorously chopped (up to 30 s) [20] in isolation buffer with a fresh razor blade. Three biological replicates comprised of five axes or one leaf fragment were utilized in the assay of each treatment condition. Several variants of isolation buffer were evaluated A) 0.4 mM Tris pH 7.4; B) PBS (137 mM NaCl, 2.7 mM KCl, 10 mM Na_2_HPO_4_, 1.8 mM KH_2_PO_4_ and KH_2_PO_4_; and C) 50 mM Sörensen buffer pH 6.8 (51% NaH_2_PO, 49% Na_2_HPO_4_). The buffers were supplemented with Na_2_EDTA and DMSO to a final concentration of 0.5 mM and 0.5%, respectively. The plates were kept tilted on ice for 10 min allowing the isolated nuclei to be collected in the buffer. The suspension was subsequently transferred to 1.5 mL Eppendorf tubes and kept at 4 °C for another 10 min to separate the nuclei from the cellular debris. The nuclear suspension (100 μl) was then gently mixed with 1% low melting-point agarose (LMP; TopVison Low Melting Point Agarose; Thermo Fisher Scientific, Waltham, MA, USA) pre-warmed to 42 °C. Subsequently, 50 μl aliquots were placed on three microscope slides pre-coated with 1% normal melting-point agarose (NMP agarose) for 1) the alkaline comet assay; 2) the enzyme-modified version (+ FPG; formamidopyrimidine DNA glycosylase; or 3) the buffer control for the enzyme-modified version (− FPG). The drops of nuclei suspension were then covered with a coverslip, placed on a cold chilling plate, and incubated at 4 °C for 10 min. The coverslips were then removed and the slides were placed in vertical slide carriers that allowed the simultaneous manipulation of 25 slides each, which were then placed in an ebony treatment dish for subsequent lysis. Although the time for lysis is not believed to be critical to the final outcome, it has been suggested that it should not be shorter than 1 h [5]. Therefore, the slides were left in the lysis buffer (2.5 M NaCl, 100 mM Na_2_EDTA, 1% Triton X-100, 10 mM Tris, pH 10) for 1 h at 4 °C to remove histones, disrupt nucleosomes and the nuclear membrane, and prepare the resulting nucleoids [17, 25]. DMSO was added to a variant of the lysis solution to a final concentration of 10% [20]. Slides for the enzyme-modified comet assay (−/+ FPG) were washed three times for 5 min with FPG buffer (50 mM Tris-HCL, 2 mM Na_2_EDTA, KCl 50 mm, pH 7.5). Next, 50 μL of FPG enzyme (diluted 1:3000; 0.6 U/mL) in a FPG reaction buffer supplemented with BSA (0.2 mg/mL) were added to the gel and the slides were covered with parafilm. Only FPG reaction buffer was added on the control slides (−FPG) to allow the determination of net incisions [72]. The slides were incubated at 37 °C for 30 min [73] in a humidified box. Afterwards, slides were rinsed two times with 0.4 M Tris HCl pH 7.5 and placed in a dish containing a freshly-prepared, cold, alkaline solution (1 mM Na_2_EDTA, 300 mM NaOH, pH > 13) at 4 °C for 40 min [16] to allow DNA to unwind prior to electrophoresis in a vertical tank (20 min at 1 V/cm). The electrophoretic solution was kept cold with the use of cooling packs. Neutralization was performed using 0.4 M Tris-HCl, pH 7.5 twice for 5 min and then the slides were dehydrated in methanol for 10 min and dried overnight at RT. The slides were stained with SybrGold (dilution: 1:10,000 in 1x PBS, Invitrogen, Waltham, MA, USA) for 1 h and washed in dd H_2_O for 10 min prior to imaging. Typically, 50 randomly selected comets, in the three biological replicates collectively, were scored using a fluorescent microscope (Leica TCS SP5 II, Germany), and the level of DNA strand breaks (% tail DNA) of the comets was recorded using Comet Assay IV analysis software (Perceptive Instruments, Haverhill, Suffolk, UK) (Supplementary file Fig. S3). Embryonic axes treated with H_2_O_2_ in the range 0.01–0.25 mM, or subjected to accelerating-ageing conditions, were used as positive controls. Importantly, in the in vitro viability assessment, embryonic axes were kept for 18 h at 4 °C on moistened filter paper prior to sterilization to avoid damage resulting from excessive absorption of the sterilization solution. For these reasons, the potential of DNA repair after a short time (1 h) and a longer time (18 h) following desiccation was assessed in the present study.

### DNA isolation and assessment of DNA integrity

Total genomic DNA was extracted from embryonic axes after homogenization of the tissue in LN using NucleoSpin Plant II Kit (Macherey-Nagel, Düren, Germany) according to the manufacturer’s instructions. Each experiment was replicated three times and five embryonic axes were used in each replicate. DNA concentration and quality (A_260_/A_280_ = 1.8–1.9) was measured with a NanoQuant Plate M200 PRO (Tecan, Männedorf, Switzerland). DNA integrity was analyzed using an automated electrophoresis platform TapeStation 4200 (Agilent Technologies, Santa Clara, CA, USA) with Genomic DNA ScreenTape (sizing range 200 to > 60,000 bp) or D1000 ScreenTape (sizing range 35–1000 bp) according to the manufacturer’s instructions. This system provides a numerical measure of genomic DNA integrity (DNA Integrity Number, DIN) ranging from 1 to 10, where 1 indicates highly degraded DNA and 10 represents highly intact DNA.

### Statistical analysis

R software (R Core Team 2020; https://www.r-project.org) was used for the statistical analyses and graphical visualization of data. The effect of desiccation on viability, as measured by TTC staining assay, survival, and regrowth, were separately evaluated using a general linear model (GLM) with a binomial distribution. The impact of desiccation on the level of ROS, ^٠^OH, DNA strand breaks, 8-oxoG and DNA fragmentation was also evaluated using a linear model. The assessment of normality of the data was conducted using the Shapiro-Wilk test. The Lambert W x F transformation was applied to non-normally distributed data of the % DNA in the tail and ^٠^OH level. A one-way ANOVA was used to test significant differences between the mean values. Pairwise comparisons between treatments were performed with the application of Duncan’s multiple range test at *p* ≤ 0.05. An R-package ‘corrplot‘ was used for determining the correlation between measured traits. R-packages ‘ggplot2’, ‘factoextra‘ and ‘FactoMineR’ were used for the principal component analysis (PCA). The R-package ‘ggplot2’ was used for graphical visualization of the data.

## Supplementary Information

**Additional file 1: Fig. S1.** The impact of increasing concentrations of H_2_0_2_ on formation of comets from nuclei embedded in LMP agarose in the alkaline comet assay.

**Additional file 2: Fig. S2.** The influence of gradual desiccation and accelerating ageing treatment on *Acer pseudoplatanus* L. embryonic axes DNA fragmentation analyzed by automated electrophoresis. Analysis was repeated three times, representative histograms are presented.

**Additional file 3: Fig. S3.** The representative comet measurements and images captured by Comet Assay IV analysis software. The blue line represents the start of the head, the green line is the middle of the head and the purple line is the end of the tail. The fluorescence intensity in the comet tail indicates the extent of DNA damage.

**Additional file 4: Table S1 and S2.** Table of coordinates of variables used in principal component analysis.

## Data Availability

The datasets supporting the conclusions of this article are included within the article and its supplementary materials published online or are available from the corresponding author on request.

## Abbreviations

ALS: alkali-labile sites
BER: base excision repair
DIN: DNA Integrity Number
FaPyG: 2,6-diamino-4-hydroxy-5-formamidopyrimidine
FPG: formamidopyrimidine DNA glycosylase
H_2_DCFDA: 2′,7′-dichlorodihydrofluorescein diacetate
H_2_O_2_: hydrogen peroxide
MC: moisture content
NER: nucleotide excision repair
^٠^OH: hydroxyl radical
8-oxoG: 8-oxo-7,8-dihydroguanine
RFU g^− 1^ DW: fluorescence units per gram of dry weight
ROS: reactive oxygen species
TTC: 2,3,5-triphenyltetrazolium chloride
WC: water content
