# Supplementary material for: Oxidative damage and DNA repair in desiccated recalcitrant embryonic axes of Acer pseudoplatanus L
Source: BMC Plant Biol. 2022 Jan 19;22:40. doi: 10.1186/s12870-021-03419-2 (PMC8767751; doi:10.1186/s12870-021-03419-2)

Figure S1. The impact of increasing concentrations of H_20_2 on formation of comets from nuclei embedded in LMP agarose in the alkaline comet assay

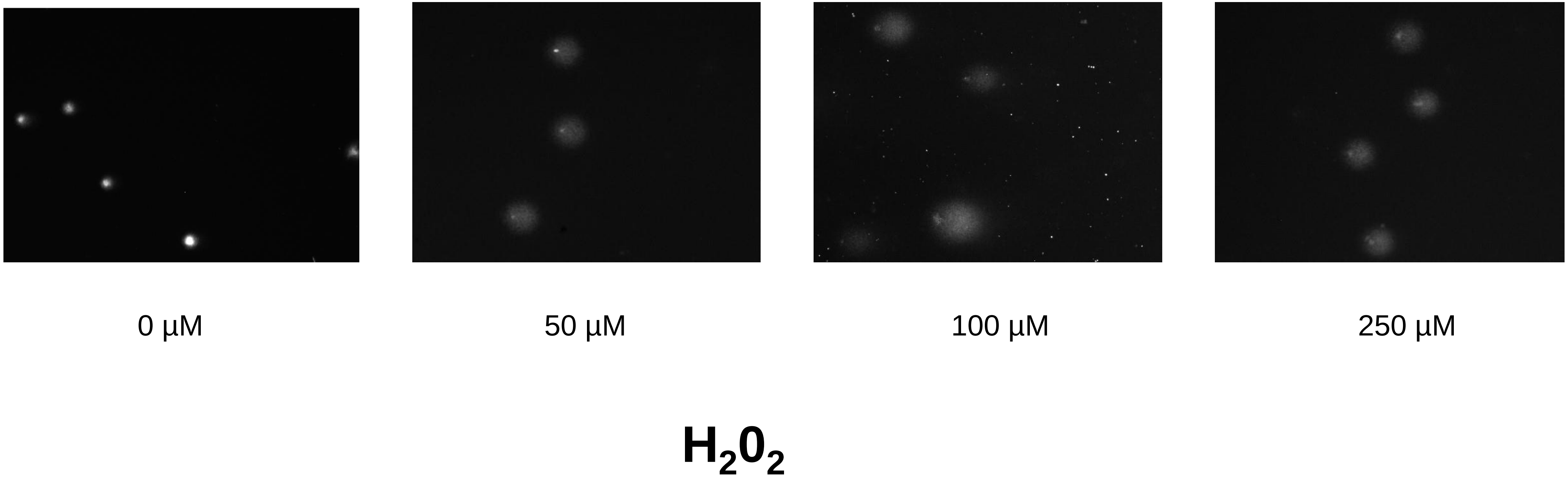

Supplement: Supplementary file 1 — Additional file 1: Fig. S1. The impact of increasing concentrations of H202 on formation of comets from nuclei embedded in LMP agarose in the alkaline comet assay. [file 12870_2021_3419_MOESM1_ESM.pdf]
