# Supplementary material for: Oxidative damage and DNA repair in desiccated recalcitrant embryonic axes of Acer pseudoplatanus L
Source: BMC Plant Biol. 2022 Jan 19;22:40. doi: 10.1186/s12870-021-03419-2 (PMC8767751; doi:10.1186/s12870-021-03419-2)

Figure S2A. The influence of gradual desiccation and accelerating ageing at 45°C for 72h of *Acer pseudoplatanus L.* embryonic axes on DNA fragmentation analysed by automated electrophoresis in a range from 200 - > 60,000 bp. Analysis was repeated three times, representative histograms are presented.

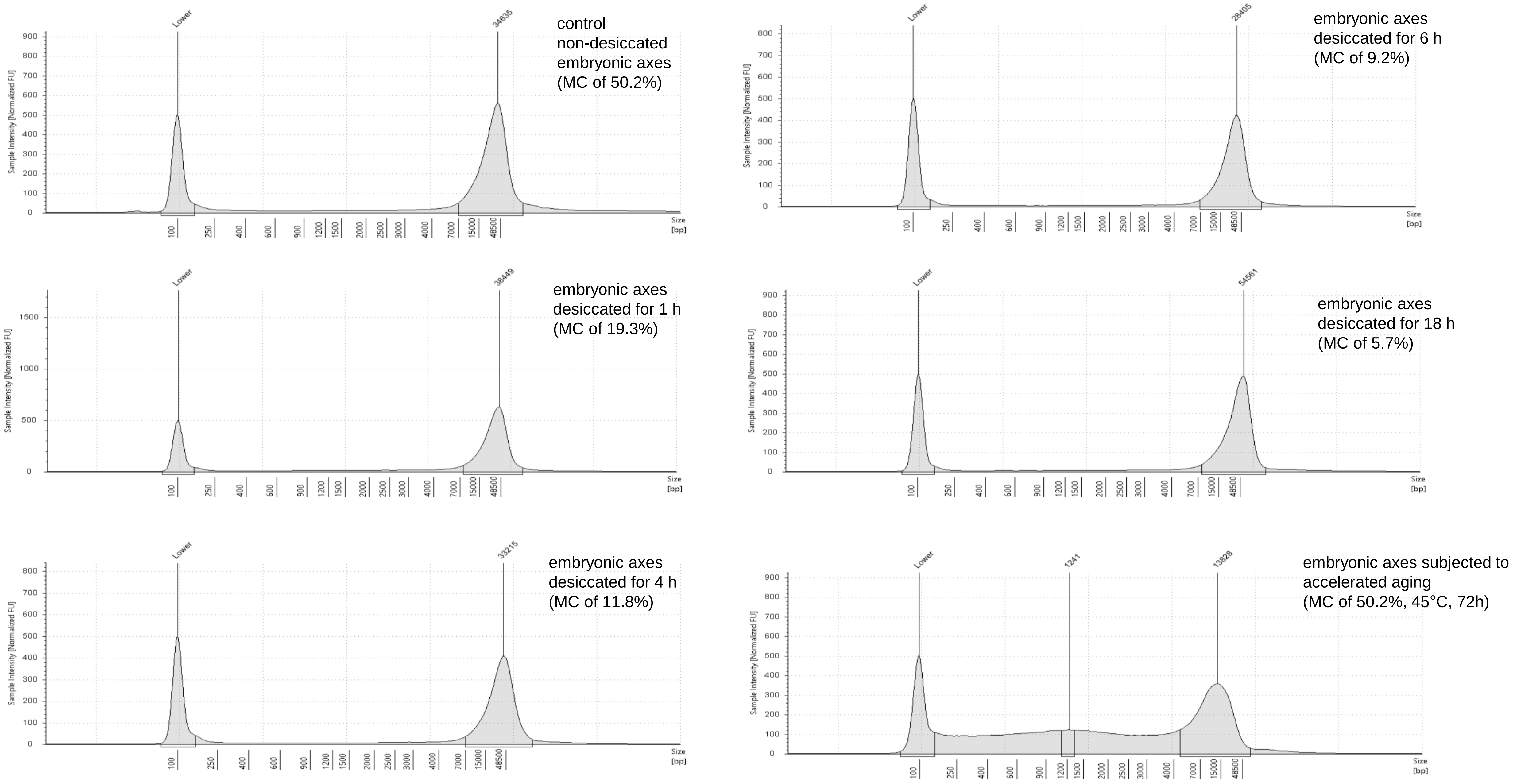

Figure S2B. The influence of gradual desiccation and accelerating ageing at 45°C for 72h of *Acer pseudoplatanus L.* embryonic axes on DNA fragmentation analysed by automated electrophoresis in a range from 35-1000 bp. Analysis was repeated three times, representative histograms are presented.

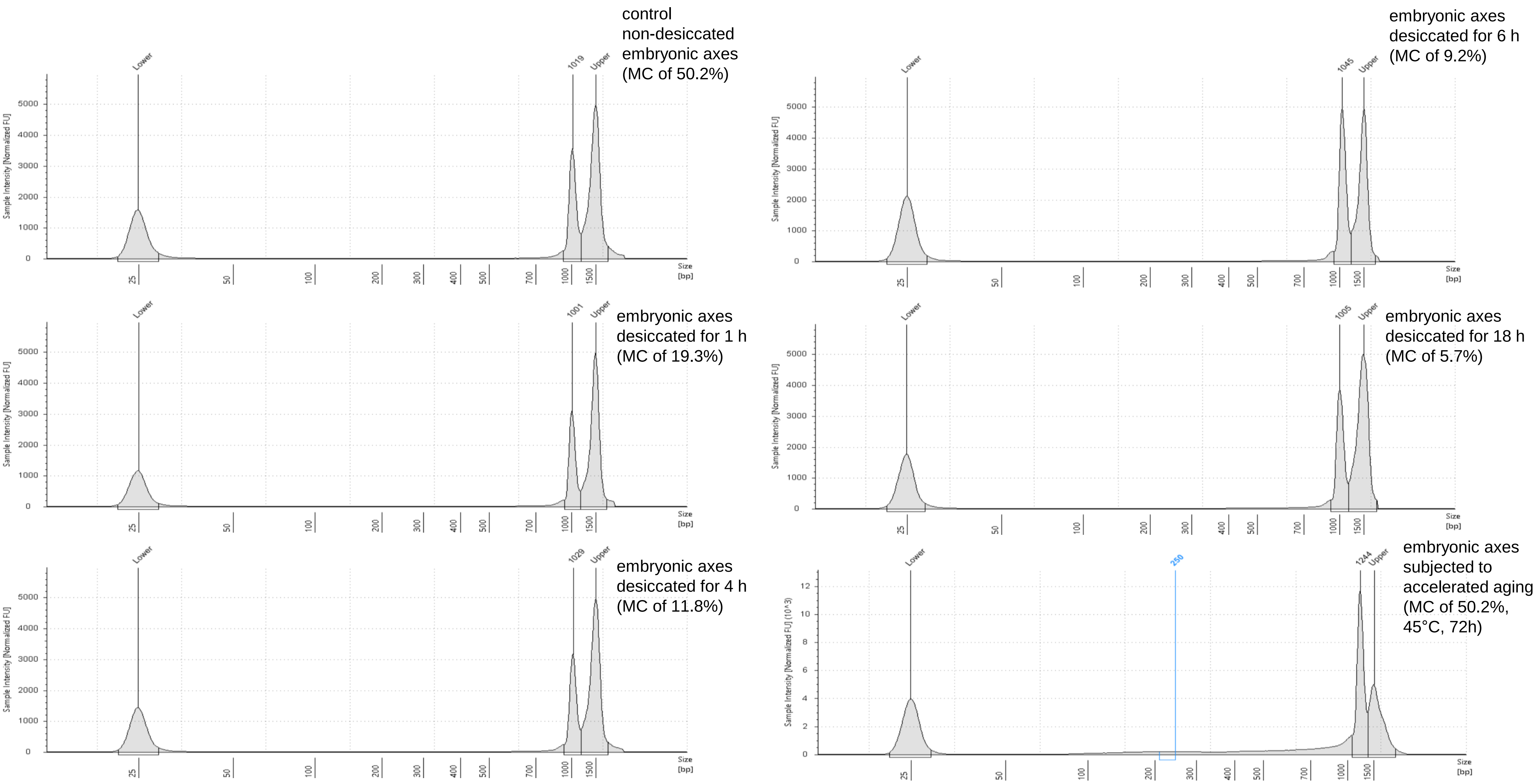

Supplement: Supplementary file 2 — Additional file 2: Fig. S2. The influence of gradual desiccation and accelerating ageing treatment on Acer pseudoplatanus L. embryonic axes DNA fragmentation analyzed by automated electrophoresis. Analysis was repeated three times, representative histograms are presented. [file 12870_2021_3419_MOESM2_ESM.pdf]
