# Supplementary material for: Oxidative damage and DNA repair in desiccated recalcitrant embryonic axes of Acer pseudoplatanus L
Source: BMC Plant Biol. 2022 Jan 19;22:40. doi: 10.1186/s12870-021-03419-2 (PMC8767751; doi:10.1186/s12870-021-03419-2)

Figure S3. The representative comet measurements and images captured by Comet Assay IV analysis software

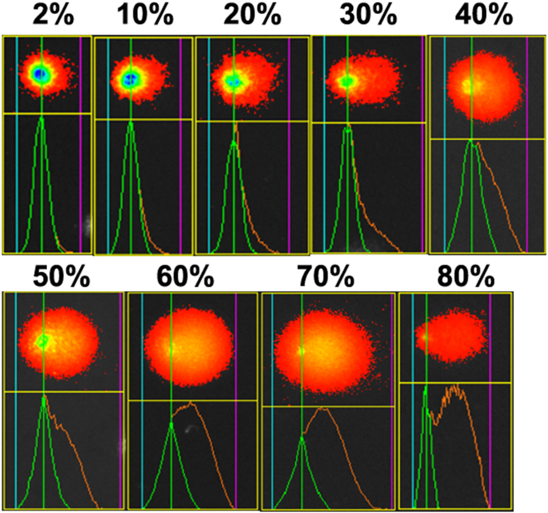

Supplement: Supplementary file 3 — Additional file 3: Fig. S3. The representative comet measurements and images captured by Comet Assay IV analysis software. The blue line represents the start of the head, the green line is the middle of the head and the purple line is the end of the tail. The fluorescence intensity in the comet tail indicates the extent of DNA damage. [file 12870_2021_3419_MOESM3_ESM.pdf]
