# Supplementary material for: Oxidative damage and DNA repair in desiccated recalcitrant embryonic axes of Acer pseudoplatanus L
Source: BMC Plant Biol. 2022 Jan 19;22:40. doi: 10.1186/s12870-021-03419-2 (PMC8767751; doi:10.1186/s12870-021-03419-2)
Supplement: Supplementary file 4 — Additional file 4: Table S1 and S2. Table of coordinates of variables used in principal component analysis. [file 12870_2021_3419_MOESM4_ESM.pdf]

Table S1. Table of coordinates of variables used in principal component analysis.

Variable	Dim.1	Dim.2	Dim.3	Dim.4	Dim.5	Dim.6	Dim.7	Dim.8	Dim.9
OH	0.330101	0.556496	0.400577	-0.6365	-0.06074	0.046031	-0.09337	-0.03324	0.002457
ROS	0.372993	-0.82509	0.158458	0.118255	-0.37257	0.014512	-0.02419	-0.03046	-0.01707
TTC	-0.91159	-0.18703	0.33096	-0.00566	-0.03977	-0.07723	-0.08761	0.084264	-0.00949
Regrow.	-0.95233	-0.12245	0.221301	-0.14372	-0.0557	-0.00778	0.049133	0.041487	0.009398
Surviv.	-0.92786	-0.1417	0.291107	-0.15712	-0.00051	-0.01161	0.084288	0.001083	0.026242
SB t0h	0.947885	0.206363	-0.14778	0.00716	-0.01551	-0.16654	-0.03352	0.070637	-0.0125
SB t1h	0.788628	0.256269	0.506139	0.154955	0.021842	0.146172	0.070649	0.059862	-0.04224
SB t18h	0.77465	0.292626	0.503625	0.165917	-0.06116	-0.1482	0.062255	-0.02869	0.043112
8-oxoG t0h	0.368346	-0.79564	0.152868	-0.39071	0.20151	-0.09878	0.044898	-0.02871	-0.04401
8-oxoG t1h	0.742599	-0.28728	-0.3716	-0.45121	-0.10683	0.053645	0.058814	0.066135	0.038346
8-oxoG t18h	0.58684	-0.70295	0.275465	0.169809	0.203138	0.076234	-0.08078	0.015652	0.050597

Table S2. Table of contribution of variables used in principal component analysis.

Variable	Dim.1	Dim.2	Dim.3	Dim.4	Dim.5	Dim.6	Dim.7	Dim.8	Dim.9
OH	1.816428	12.5679	13.39627	44.95657	1.506092	2.162109	17.97971	4.300213	0.055212
ROS	2.319131	27.62778	2.096232	1.551802	56.67399	0.214884	1.207259	3.610468	2.665988
TTC	13.8524	1.419531	9.144588	0.00356	0.645836	6.08602	15.82931	27.63376	0.824386
Regrow.	15.11804	0.608474	4.088635	2.292179	1.266726	0.061798	4.978748	6.698531	0.808104
Surviv.	14.3512	0.814893	7.074847	2.739365	0.000105	0.137634	14.65204	0.004568	6.300418
SB t0h	14.97739	1.728244	1.823319	0.005688	0.098264	28.30129	2.316612	19.41885	1.428769
SB t1h	10.36739	2.665219	21.38709	2.66447	0.194779	21.80245	10.29385	13.94643	16.32026
SB t18h	10.00312	3.475075	21.17515	3.054785	1.527404	22.4112	7.993098	3.2039	17.00456
8-oxoG t0h	2.261705	25.69022	1.950942	16.93993	16.57918	9.95581	4.157458	3.207268	17.71716
8-oxoG t1h	9.192512	3.349224	11.52795	22.59184	4.659606	2.936566	7.13388	17.02257	13.45295
8-oxoG t18h	5.740689	20.05344	6.334973	3.199805	16.84802	5.930242	13.45803	0.953433	23.4222
